# Adjustment of the GRACE score by the triglyceride glucose index improves the prediction of clinical outcomes in patients with acute coronary syndrome undergoing percutaneous coronary intervention

**DOI:** 10.1186/s12933-022-01582-w

**Published:** 2022-08-05

**Authors:** Shiqiang Xiong, Qiang Chen, Xu Chen, Jun Hou, Yingzhong Chen, Yu Long, Siqi Yang, Lingyao Qi, Hong Su, Wenchao Huang, Hanxiong Liu, Zhen Zhang, Lin Cai

**Affiliations:** Department of Cardiology, The Third People’s Hospital of Chengdu, Affiliated Hospital of Southwest Jiaotong University, Chengdu, 610014 Sichuan China

**Keywords:** GRACE score, The triglyceride-glucose index, Acute coronary syndrome, Insulin resistance, Fasting blood glucose, Percutaneous coronary intervention, Major adverse cardiac events, Residual SYNTAX score, Prognosis

## Abstract

**Background:**

The Global Registry of Acute Coronary Events (GRACE) score derived from clinical parameters at the time of hospital discharge is a powerful predictor of long-term mortality and reinfarction after acute coronary syndrome (ACS). The triglyceride glucose (TyG) index, which is a simple and reliable surrogate marker of insulin resistance, has been demonstrated to be an independent predictor of long-term adverse major adverse cardiac events, irrespective of diabetes mellitus. We investigate whether the addition of the TyG index improves the predictive ability of the GRACE score after percutaneous coronary intervention (PCI) in ACS patients regardless of diabetes mellitus.

**Method:**

A retrospective cohort of 986 ACS patients undergoing PCI was enrolled in the present analyses. The GRACE score for discharge to 6 months and the TyG index were calculated. The primary endpoint was the composite of MACEs, including all-cause death and nonfatal myocardial infarction. Patients were stratified according to the primary endpoint and the tertiles of the TyG index. Cumulative curves were calculated using the Kaplan–Meier method. Multivariate Cox regression was adopted to identify predictors of MACEs. The predictive value of the GRACE score alone and combined with the TyG index or fasting blood glucose (FBG) was estimated by the area under the receiver‑operating characteristic curve, likelihood ratio test, Akaike’s information criteria, continuous net reclassification improvement (NRI), and integrated discrimination improvement (IDI). Internal validation was assessed using the means of bootstrap method with 1000 bootstrapped samples.

**Results:**

During a median follow-up of 30.72 months ((interquartile range, 26.13 to 35.07 months), 90 patients developed MACEs, more frequently in the patients with a higher TyG index. Multivariate Cox hazards regression analysis found that the TyG index, but not FBG was an independent predictor of MACEs (hazard ratio 1.6542; 95% CI 1.1555–2.3681; P = 0.006) in all types of ACS regardless of diabetes mellitus when included in the same model as GRACE score. Furthermore, Kaplan–Meier analysis revealed that the incidence of the primary endpoint rose with increasing TyG index tertiles (log-rank, P < 0.01). Adjustment the GRACE score by the TyG index improved the predictive ability for MACEs (increase in C-statistic value from 0.735 to 0.744; NRI, 0.282, 95% CI 0.028–0.426, P = 0.02; IDI, 0.019, 95% CI 0.004–0.046, P = 0.01). Likelihood ratio test showed that the TyG index significantly improved the prognostic ability of the GRACE score (χ^2^ = 12.37, 1 df; P < 0.001). The results remained consistent when the models were confirmed by internal bootstrap validation method.

**Conclusion:**

The TyG index, but not FBG is an independent predictor of long-term MACEs after PCI in all types of ACS patients regardless of diabetes mellitus after adjusting for the GRACE score, and improves the ability of the GRACE score to stratify risk and predict prognosis of ACS patients undergoing PCI.

**Supplementary Information:**

The online version contains supplementary material available at 10.1186/s12933-022-01582-w.

## Introduction

Patients with acute coronary syndrome (ACS) present with diverse clinical characteristics and risk of adverse cardiovascular outcomes. The need to risk stratify ACS patients at discharge for long-term prognosis is widely accepted, which is helpful for making appropriate management decisions. The Global Registry of Acute Coronary Events (GRACE) score derived from clinical parameters at the time of hospital discharge is a powerful predictor of long-term mortality and reinfarction in all types of ACS patients [1–3]. However, some validated predictors associated with unfavorable prognosis have not been included as variables in the GRACE scoring system. Therefore, it is necessary to investigate whether a combination of other prognostic factors with the GRACE score provides a more accurate prognostic assessment and precise yet user-friendly risk stratification in patients with ACS.

The triglyceride glucose (TyG) index, which is a simple, cost-effective, reliable surrogate marker of insulin resistance, has been demonstrated to be associated with an increased risk of cardiovascular diseases in the general population [4–7], and an independent predictor of adverse cardiovascular outcomes after percutaneous coronary intervention (PCI) in different cohorts, irrespective of diabetes [8–13]. To the best of our knowledge, no relevant study has focused on whether the addition of the TyG index improves the predictive ability of the GRACE score in patients with ACS undergoing PCI.

In this study, we investigated the predictive value of the TyG index in addition to GRACE score for major adverse cardiac events (MACEs) after PCI in patients with ACS and the potential incremental prognostic value of adding the TyG index to the GRACE score.

## Methods

### Study cohort

We performed a single-center, prospective observational cohort study of consecutive patients with ACS undergoing PCI in the Third People’s Hospital of Chengdu (Sichuan, China) from July 2018 to December 2020. Patients were excluded if (1) they had a history of coronary artery bypass grafting; (2) they had valvular disease and severe mechanical complications requiring cardiac surgery; (3) they had severe hepatic and renal insufficiency (creatinine clearance < 15 ml/min); (6) they suffered from malignant tumors; or (7) they died in hospital. Patients with incomplete key variables including the TyG index variables and GRACE score variables were also excluded. A total of 26 patients were excluded because of missing follow-up data despite at least four separate attempts to contact them. Ultimately, 986 patients were included in the final analyses. This study was approved by the ethics committee of the Third People’s Hospital of Chengdu and strictly complied with the Declaration of Helsinki, with a waiver of patient informed consent. Personal information related to the identities of the participants was concealed.

### Data collection and definitions

Data on demographics, pervious medical history, smoking, laboratory examination, and medical and procedural information of participants at admission and during hospitalization were obtained from the electronic medical records. Pervious medical histories, such as PCI, chronic obstructive pulmonary disease, hypertension, diabetes mellitus, stroke, and atrial fibrillation were obtained from self-reported information and then confirmed by corresponding medical records. Self-reported diabetes mellitus with the use of antidiabetic medication before hospitalization, or the symptoms of diabetes mellitus with casual blood glucose ≥ 11.1 mmol/L, fasting blood glucose (FBG) ≥ 7.0 mmol/L, and/or 2-h blood glucose ≥ 11.1 mmol/L in the 75 g oral glucose tolerance test were the diagnostic criteria for diabetes mellitus [14]. Hypertension was defined as at least three blood pressure measurements ≥ 140/90 mmHg [15], and/or currently receiving antihypertensive treatments. ACS was defined as including either unstable angina (UA), ST segment elevation myocardial infarction (STEMI), or non-ST segment elevation myocardial infarction (NSTEMI) [16].

Peripheral venous blood was sampled from patients after overnight fasting (> 8 h). Venous plasma concentrations of total cholesterol (TC), triglycerides (TG), low-density lipoprotein-C (LDL-C), high-density lipoprotein-C (HDL-C), FBG, serum creatinine, brain natriuretic peptide (BNP), and cardiac troponin T (cTnT) were determined by standard biochemical techniques. The two-dimensional modified Simpson’s method was used to determine the left ventricular ejection fraction (LVEF).

### Calculation of the TyG index, GRACE score, and baseline and residual SYNTAX score

In brief, the TyG index is derived from fasting TG and FBG, and was calculated as: ln [fasting TG (mg/dL) ×FBG (mg/dL)/2] [17]. The web-based GRACE score calculator was used to calculate the risk of 6-month mortality or MI for each individual [1]. The GRACE score was originally designed to calculate the risk of mortality or MI from discharge to 6 months and has been shown to provide good discrimination of mortality and MI up to 4 years after an ischemic event [2, 18]. A web-based online calculation tool (http://syntaxscore.com/) was used to calculate the baseline SYNTAX score (bSS) from the preprocedural angiograms by two independent operators who were blinded to baseline clinical characteristics and clinical outcomes. The residual SYNTAX score (rSS) was calculated based on other obstructive coronary disease cases after treatment with PCI [19]. In the case of staged PCI procedures, the rSS after the last planned revascularization was used as the entry point for final analysis. In case of disagreement, the opinion of the third operator was obtained and the decision was made by consensus. A level of rSS greater than 8 was defined as incomplete revascularization. All data were entered into a dedicated computer database and assessed for quality.

### Follow-up and endpoints

Clinical follow-up was scheduled at 1, 3, 6, and 12 months, and then every 6 months after hospital discharge by telephone contact or outpatient clinical visits. Follow-up clinical events were investigated and recorded by well-trained professionals. The primary endpoint was major adverse cardiac events (MACEs), defined as a composite of all-cause death and nonfatal MI. Secondary endpoints included cardiac death, unplanned revascularization, and nonfatal stroke. All clinical endpoints were further confirmed by referring to corresponding medical records as necessary. All-cause death referred to death regardless of the cause. MI was defined as elevated creatine kinase or cardiac troponin greater than the upper limit of the normal range with ischemia indicated from electrocardiographic changes and/or symptoms. Unplanned revascularization was defined as any ischemia-driven target or nontarget revascularization after the index PCI during the follow-up period. Stroke was defined as an ischemic or hemorrhagic stroke as demonstrated by the evidence of neurological dysfunction or clinically documented lesions on imaging.

### Statistical analysis

Categorical variables are presented as numbers and percentages. Continuous variables are expressed as the mean and SD or median and interquartile range. ANOVA test or the Kruskal-Wallis test was used as appropriate. Study participants were stratified into three groups based on the TyG index tertiles. The cumulative incidence of clinical outcomes was assessed using the Kaplan-Meier method and determined by the log-rank test. The hazard ratio (HR) with 95% confidence interval (CI) of developing the primary endpoint was estimated by the Cox proportional regression model. Both the TyG index and GRACE score were analyzed as continuous variables. Variables identified through univariate analysis with P values < 0.10 were selected for multivariate analysis.

The χ^2^ likelihood ratio tests were used to estimate whether the addition of the TyG index or FBG to the logistic regression model that included the GRACE score provided a significantly better fit. Comparison of nested and non-nested models including the GRACE score, or its combination with the TyG index or FBG was performed by calculating corrected Akaike’s information criterion (AICc), delta-AICc (δAICc), and AICc weights (AICcWt) to determine the adequacy that a given model was the best fitting one of those studied [20].

The optimism for different models was determined by internal validation using the means of bootstrap method (with 1000 bootstrapped samples) with the relatively corrected C-index. The differences in performance indicated the expected optimism, which were calculated by subtracting the optimism from the apparent model performance [21]. Calibration curves, which describe the calibration of the model in terms of the agreement between the predicted risks of adverse cardiovascular events and observed frequency of adverse cardiovascular events, were also adopted to estimate the model performance [22]. The y-axis represents the actual adverse cardiovascular events rate. The x-axis represents the predicted adverse cardiovascular events risk. The grey line indicates a perfect prediction by an ideal model. The red solid line indicates the performance of the predicting model, of which a closer fit to the grey line suggests better prediction.

The area under the receiver operating characteristic (ROC) curves was adopted to identify the predictive value of different parameters or risk models for clinical outcomes. The incremental predictive value from adding the TyG index or FBG to the GRACE score to predict clinical outcomes was analyzed from these predicted probabilities using several measures of improvement in discrimination: C-statistics, net reclassification improvement (NRI) and integrated discrimination improvement (IDI). All tests were 2-sided, and P < 0.05 was considered statistically significant.

All statistical analyses were conducted using SPSS version 26.0 software (IBM Corporation, Chicago, IL, USA) and R version 4.0.2 software (Vienna, Austria).

## Results

### Baseline characteristics

A total of 986 patients (66.61 ± 11.42 years, 28.3% female) who presented with ACS and completed the follow-up were available for the final analysis. During a median follow-up of 30.72 months (IQR, 26.13 to 35.07 months), there were 90 MACEs (9.13%). The baseline characteristics of the total population are summarized in Table 1. All patients were stratified into three groups [T1 (TyG index ≤ 8.65), T2 (8.65 < TyG index ≤ 9.24), and T3 (TyG index > 9.24)] in accordance with tertiles of the TyG index (Table 1). The mean TyG index values of the three groups were 8.28 ± 0.29, 8.96 ± 1.73, and 9.79 ± 0.47, respectively. Compared with patients in the tertile 1 group, patients with a higher TyG index tended to be younger, female, had a higher prevalence of diabetes mellitus, ST-segment depression, multivessel disease and incomplete revascularization, higher levels of body mass index, FBG, TG, TC, LDL-C, bSS, and rSS, a lower level of HDL-C, and used more insulin and oral hypoglycemic agents at discharge.

**Table 1:** Baseline characteristics of the patients stratified by the TyG index tertiles

Variable	T1(n = 332)	T2 (n = 327)	T3 (n = 327)	P value
*GRACE variables*				
Age, years	68.10 ± 11.89	66.65 ± 10.95	65.07 ± 11.24	0.003
SBP, mmHg	131.28 ± 19.81	133.17 ± 22.69	132.25 ± 21.66	0.525
h, bpm	76.15 ± 14.41	78.15 ± 14.95	78.66 ± 14.96	0.071
Serum creatinine, umol/L	77.60 (65.55, 90.75)	78.10 (66.80, 92.70)	73.20 (61.10, 90.20)	0.046
CHF, n (%)	125 (37.7)	128 (39.1)	142 (43.4)	0.292
Previous MI, n (%)	15 (4.5)	21 (6.4)	17 (5.2)	0.548
ST-segment depression, n (%)	129 (38.9)	124 (37.9)	155 (47.4)	0.025
Elevated cardiac enzymes/markers, n (%)	210 (63.3)	221 (67.6)	232 (70.9)	0.108
*GRACE score*	110.61 ± 31.99	109.54 ± 29.91	109.30 ± 32.31	0.851
*the TyG index*	8.28 ± 0.29	8.96 ± 1.73	9.79 ± 0.47	<0.001
Female, n (%)	72 (21.7)	83 (25.4)	124 (37.9)	<0.001
BMI, kg/m^2^	23.58 ± 2.89	24.61 ± 2.78	24.77 ± 2.78	<0.001
Smoking, n (%)	181 (54.5)	187 (57.2)	173 (52.9)	0.539
Previous PCI, n (%)	30 (9.0)	29 (8.9)	24 (7.3)	0.689
COPD, n (%)	22 (6.6)	20 (6.1)	13 (4.0)	0.291
Hypertension, n (%)	208 (62.7)	217 (66.4)	214 (65.4)	0.582
Diabetes mellitus, n (%)	49 (14.8)	105 (32.1)	189 (57.8)	<0.001
AF, n (%)	27 (8.1)	20 (6.1)	19 (5.8)	0.431
Previous Stroke, n (%)	25 (7.5)	27 (8.3)	23 (7.0)	0.839
*Laboratory measurements*				
cTnT, pg/mL	28.29 (10.73, 968.00)	30.10 (11.84, 652.60)	59.25 (13.36, 1006.60)	0.233
BNP, pg/mL	109.85 (45.03, 301.08)	103.50 (32.60, 287.30)	111.60 (38.10, 324.00)	0.337
FBG (mmol/L)	5.39 ± 1.18	6.41 ± 1.82	9.19 ± 4.03	<0.001
TG (mmol/L)	0.97 ± 0.28	1.63 ± 0.44	2.96 ± 1.68	<0.001
TC (mmol/L)	4.07 ± 1.07	4.50 ± 1.24	4.85 ± 1.23	<0.001
HDL-C (mmol/L)	1.22 ± 0.33	1.14 ± 0.28	1.10 ± 0.27	<0.001
LDL-C (mmol/L)	2.47 ± 0.81	2.81 ± 0.92	3.22 ± 2.86	<0.001
LVEF	55.16 ± 8.81	55.33 ± 8.74	54.22 ± 9.28	0.231
*Diagnosis, n (%)*				0.262
UA	165 (49.7)	157 (48.0)	143 (43.7)	
NSTEMI	62 (18.7)	77 (23.5)	82 (25.1)	
STEMI	105 (31.6)	93 (28.4)	102 (31.2)	
*Angiographic data*				
MVD, n (%)	202 (60.8)	228 (69.7)	239 (73.1)	0.002
LM, n (%)	11 (3.3)	23 (7.0)	20 (6.1)	0.091
Calcified lesions, n (%)	42 (12.7)	44 (13.5)	50 (15.3)	0.603
Thrombosis, n (%)	20 (60.0)	28 (8.6)	31 (9.5)	0.238
Long lesion, n (%)	131 (39.5)	153 (46.8)	170 (52.0)	0.005
CTO, n (%)	54 (16.3)	82 (25.1)	67 (20.5)	0.020
Number of stents	1.35 ± 0.77	1.47 ± 0.91	1.56 ± 0.94	0.008
Length of stents, mm	35.12 ± 23.70	38.71 ± 27.56	41.51 ± 28.00	0.008
bSS	11.00 (7.00, 18.00)	14.00 (8.00, 21.00)	14.50 (8.50, 20.50)	<0.001
rSS	2.00 (0.00, 6.00)	3.00 (0.00, 7.50)	3 (0.00, 8.00)	0.010
ICR, n (%)	198 (59.6)	220 (67.3)	236 (72.2)	0.003
*Discharge medications*				
Aspirin, n (%)	322 (97.0)	320 (97.9)	319 (97.6)	0.770
P2Y12 receptor inhibitor, n (%)	328 (98.8)	324 (99.1)	322 (98.5)	0.775
Statins, n (%)	321 (96.7)	319 (97.6)	319 (97.6)	0.733
β-blockers, n (%)	218 (65.7)	231 (70.6)	238 (72.8)	0.124
ACEI/ARB, n (%)	132 (39.8)	149 (45.6)	135 (41.3)	0.295
Diuretics, n (%)	50 (15.1)	50 (15.3)	51 (15.6)	0.982
Insulin, n (%)	4 (1.2)	20 (6.1)	46 (14.1)	<0.001
Oral hypoglycemic agents, n (%)	22 (6.6)	77 (23.5)	123 (37.6)	<0.001

The patients were divided into three groups in accordance with tertiles of the TyG index [T1 (TyG index ≤ 8.65), T2 (8.65 < TyG index ≤ 9.24), and T3 (TyG index > 9.24)]. GRACE score, Global Registry of Acute Coronary Events score; TyG index, the triglyceride-glucose index; BMI, body mass index; MI, myocardial infarction; PCI, percutaneous coronary intervention; COPD, chronic obstructive pulmonary disease; AF, atrial fibrillation; SBP, systolic blood pressure; HR, heart rate; CHF, congestive heart failure; BNP, brain natriuretic peptide; FBG, fasting blood glucose; TG, triglyceride; TC, total cholesterol; HDL-C, high density lipoprotein; LDL-C, low density lipoprotein; LVEF, left ventricular ejection fraction; UA, unstable angina; STEMI, ST-segment elevation myocardial infarction; NSTEMI, non-ST-segment elevation myocardial infarction; MVD, multivessel disease; LM, left main disease; CTO, chronic total occlusion; bSS, baseline SYNTAX score; rSS, residual SYNTAX score; ICR, incomplete revascularization; ACEI/ARB, angiotensin converting enzyme inhibitor/angiotensin receptor blocker. Data are presented as the mean ± SD, median (IQR) or n (%)

### The predictive value of the TyG index and GRACE score for adverse cardiovascular events

During the 30.72-month (IQR, 26.13 to 35.07 months) follow-up, 90 (9.13%) MACEs were recorded (Table 2), including 66 (6.69%) all-cause deaths, 40 (4.06%) cardiac deaths, 26 (2.64%) nonfatal MIs, 99 (10.04%) unplanned revascularizations, and 37 (3.75%) strokes. The rate of MACEs, all-cause deaths, cardiac deaths, and unplanned revascularizations increased progressively with a higher TyG index.

**Table 2:** Comparison of long-term adverse prognosis

Variable	Total (n = 986)	T1(n = 332)	T2(n = 327)	T3(n = 327)	P value
MACEs, n (%)	90 (9.13)	20 (6.02)	27 (8.26)	43 (13.15)	0.005
All-cause death, n (%)	66 (6.69)	14 (4.22)	16 (4.89)	36 (11.01)	0.001
Cardiac death, n (%)	40 (4.06)	8 (2.41)	11(3.36)	21 (6.42)	0.025
Myocardial infarction, n (%)	26 (2.64)	6 (1.81)	11 (3.36)	9 (2.75)	0.454
Unplanned revascularization, n (%)	99 (10.04)	24 (7.23)	30 (9.17)	45 (13.76)	0.017
Stroke, n (%)	37 (3.75)	11 (3.31)	15(4.59)	11(3.36)	0.624

The patients were divided into three groups in accordance with tertiles of the TyG index [T1 (TyG index ≤ 8.65), T2 (8.65 < TyG index ≤ 9.24), and T3 (TyG index > 9.24)]. MACEs indicate major adverse cardiac events, defined as a composite of all-cause death and nonfatal myocardial infarction

**Fig. 1:**
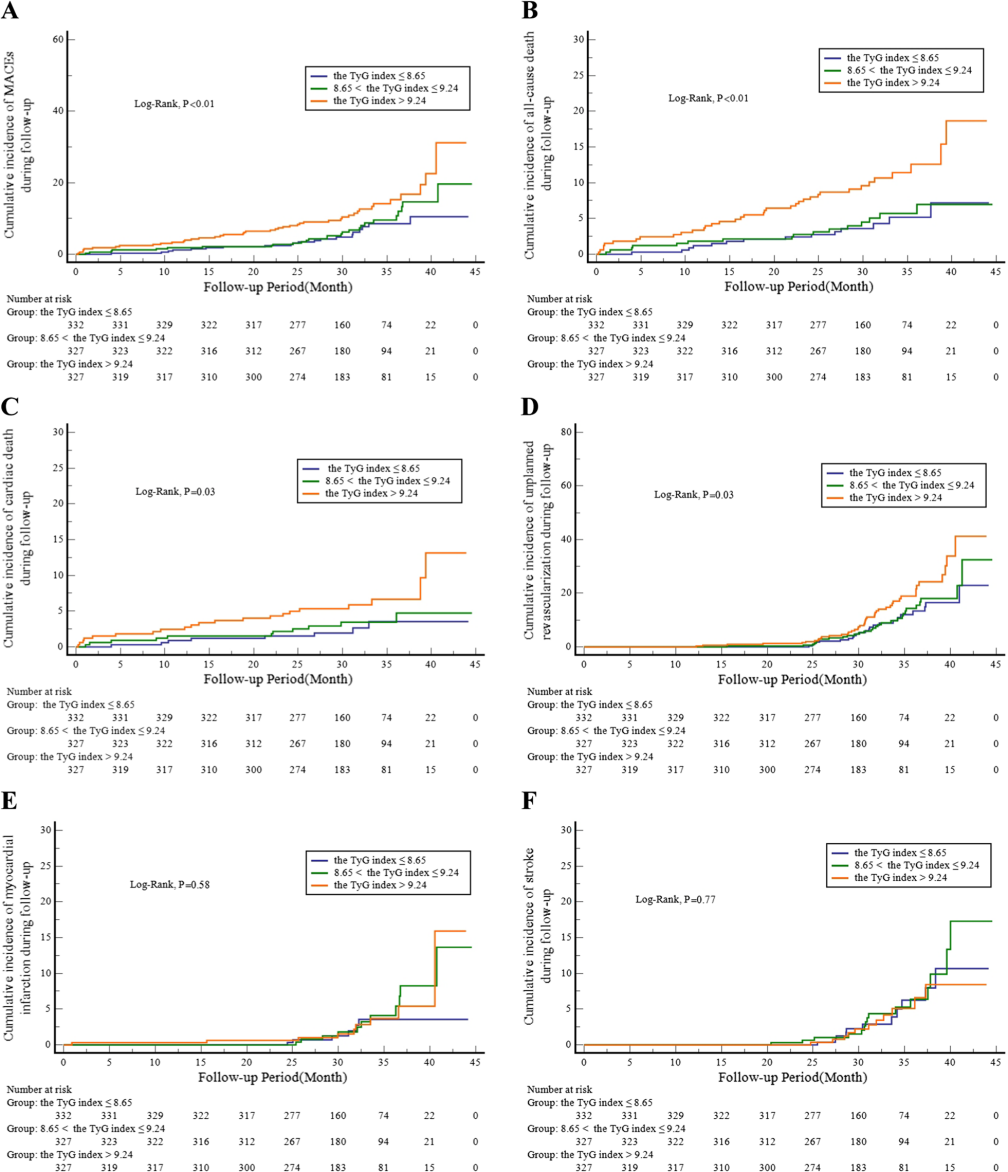
Cumulative incidence of the incidence of primary endpoint and each clinical event according to the TyG index tertiles .Kaplan-Meier curves for the incidence of MACEs (**A**), all-cause death (**B**), cardiac death (**C**), unplanned repeat revascularization (**D**), nonfatal myocardial infarction (**E**), and nonfatal stroke (**F**) among the 3 study groups based on the TyG index tertiles. TyG indicates triglyceride-glucose

Kaplan-Meier survival curves of the incidence of the primary endpoint and each clinical event for the TyG index tertiles are shown in Fig. 1. The incidence of the primary endpoint (MACEs) increased progressively with a higher TyG index (log-rank test, P < 0.01, Fig. 1A). This difference was mainly driven by the increase in all-cause deaths (log-rank test, P < 0.01, Fig. 1B). The incidence of cardiac deaths and unplanned revascularizations also increased with higher tertiles of the TyG index (Fig. 1C, D). However, the incidence of MIs and strokes at follow-up were similar among the TyG index tertiles (Fig. 1E, F).

The GRACE score, TyG index, female, bSS, ICR, LVEF, AMI, β-blocker, and diuretics were entered into the multivariate Cox regression analysis. The TyG index and GRACE score were introduced into multivariate Cox regression analysis as a continuous variable, and after adjustment for multiple confounding factors, an increased TyG index and/or GRACE score were associated with a higher risk of MACEs [HR 1.6028, (95% CI 1.1900–2.1588), P = 0.0019; 1.0202, (95% CI 1.0117–1.0288), P < 0.0001] in patients with ACS undergoing PCI (Table 3). On Cox proportional hazard regression analysis neither FBG nor TG was an independent predictor of MACEs at the final follow-up when included in the same model as GRACE score (Additional file 1: Table S1).

**Table 3:** Univariate and multivariate Cox regression analysis for predicting the primary endpoint

Variables	Univariate analysis			Multivariate analysis		
	HR	95% CI	P value	HR	95% CI	P value
GRACE score	1.0253	1.0182 to 1.0325	< 0.0001	1.0202	1.0117 to 1.0288	< 0.0001
Female	1.5832	1.0334 to 2.4255	0.0348	1.3103	0.8403 to 2.0431	0.2332
BMI	0.9528	0.8841 to 1.0268	0.2050			
Smoking	0.7895	0.5222 to 1.1936	0.2623			
Previous PCI	1.3968	0.7428 to 2.6268	0.2997			
Hypertension	1.3202	0.8409 to 2.0728	0.2274			
Diabetes mellitus	1.2838	0.8433 to 1.9544	0.2440			
TyG index	1.4961	1.1437 to 1.9571	0.0033	1.6028	1.1900 to 2.1588	0.0019
HDL-C	0.9716	0.4767 to 1.9805	0.9368			
LDL-C	0.9249	0.7572 to 1.1297	0.4441			
bSS	1.0537	1.0324 to 1.0755	< 0.0001	1.0244	0.9992 to 1.0502	0.0574
ICR	1.9863	1.2077 to 3.2669	0.0069	1.2399	0.7231 to 2.1259	0.4345
LVEF	0.9448	0.9269 to 0.9629	< 0.0001	0.9773	0.9548 to 1.0003	0.0530
AMI	1.9438	1.2533 to 3.0148	0.0030	0.8695	0.5326 to 1.4196	0.5761
β-blockers	0.9299	0.5975 to 1.4473	0.7475			
Diuretics	3.2982	2.1419 to 5.0787	< 0.0001	1.5413	0.9234 to 2.5727	0.0979
ACEI/ARB	1.2600	0.8330 to 1.9057	0.2737			
Insulin	1.4863	0.7696 to 2.8704	0.2380			

The primary endpoint was defined as a composite of all-cause death and nonfatal myocardial infarction. HR, hazard ratio; CI, confidence interval; GRACE score, Global Registry of Acute Coronary Events score; the TyG index, the triglyceride-glucose index; BMI, body mass index; FBG, fasting blood glucose; HDL-C, high density lipoprotein; LDL-C, low density lipoprotein; CTO, chronic total occlusion; ICR, incomplete revascularization; LVEF, left ventricular ejection fraction; AMI, acute myocardial infarction; bSS, baseline SYNTAX score; rSS, residual SYNTAX score; ACEI/ARB, angiotensin converting enzyme inhibitor/angiotensin receptor blocker

**Fig. 2:**
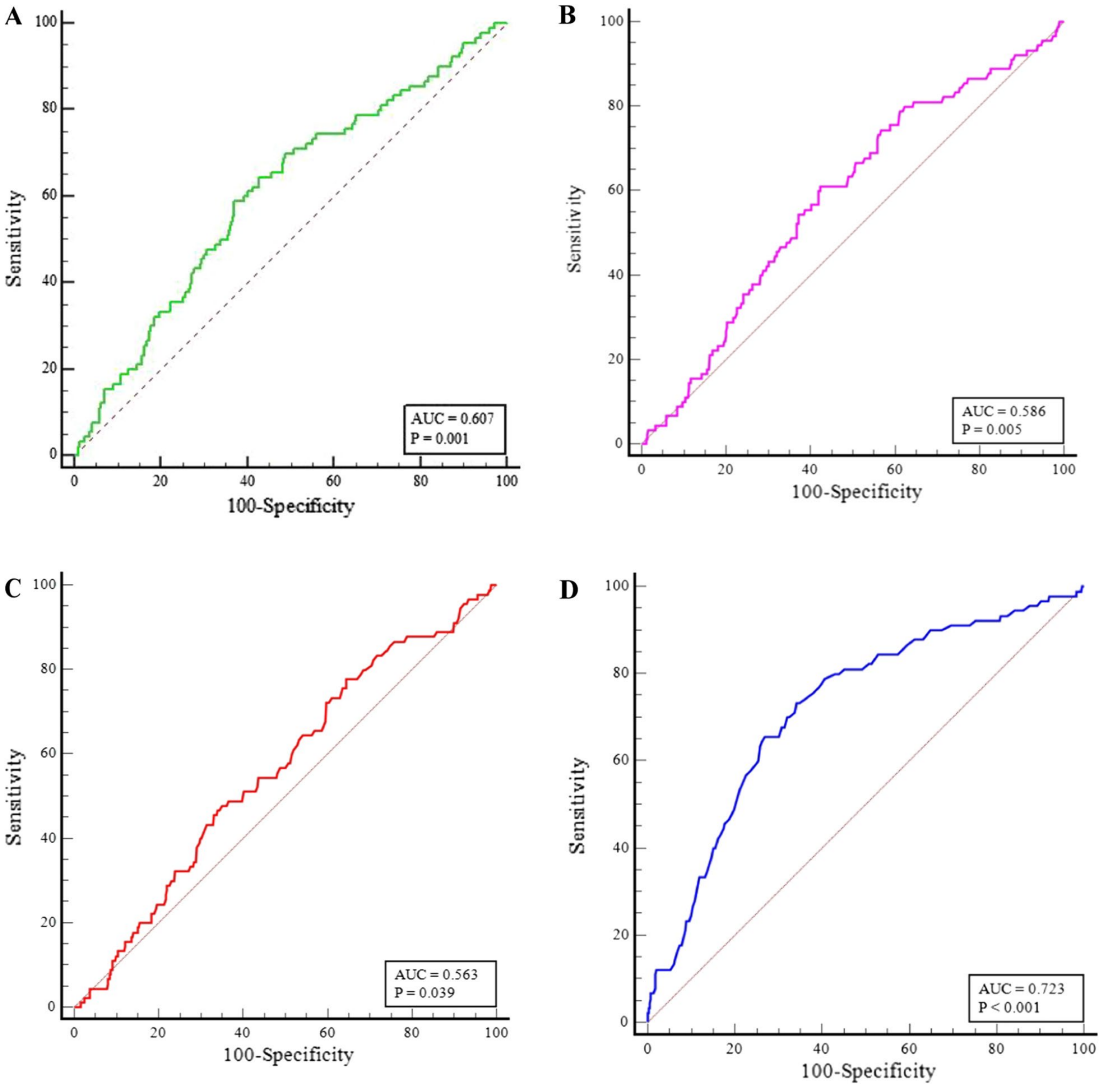
ROC curve analysis evaluating the diagnostic performance for MACEs in ACS patients undergoing PCI. **A** The area under the curve (AUC) of the TyG index for predicting MACEs was 0.607 (95% CI: 0.576–0.638, P = 0.001). **B** The AUC of the FBG for predicting MACEs was 0.586 (95% CI: 0.554–0.617, P = 0.005). The AUC of the TG (**C**) for predicting MACEs was 0.563 (95% CI: 0.532–0.594, P = 0.0039). The AUC of the GRACE score (**D**) for predicting MACEs was 0.723 (95% CI: 0.694–0.750, P < 0.001). ROC, receiver operating characteristic; ACS, acute coronary syndrome; MACEs, major adverse cardiac events; PCI, percutaneous coronary intervention; TyG, triglyceride-glucose; FBG, fasting blood glucose; TG, triglyceride

The area under the ROC curve (AUC) of the TyG index was significantly better than that of TG [0.607 (95% CI: 0.576–0.638) vs. 0.563 (95% CI: 0.532–0.594), P = 0.0182], but was not significantly greater than that of FBG [0.607 (95% CI: 0.576–0.638) vs. 0.586 (95% CI: 0.554–0.617), P = 0.4725] (Fig. 2A–C) (Additional file 1: Tables S2, S3). The AUC of the GRACE score for predicting MACEs was 0.723 (95% CI: 0.694–0.750, P < 0.001) (Fig. 2D). Taken together, we demonstrate that the TyG index, but neither FBG nor TG, independently predicts prognosis after PCI for patients with ACS after adjusting for the GRACE score.

### The predictive value of the TyG index for MACE in various subgroups

**Fig. 3:**
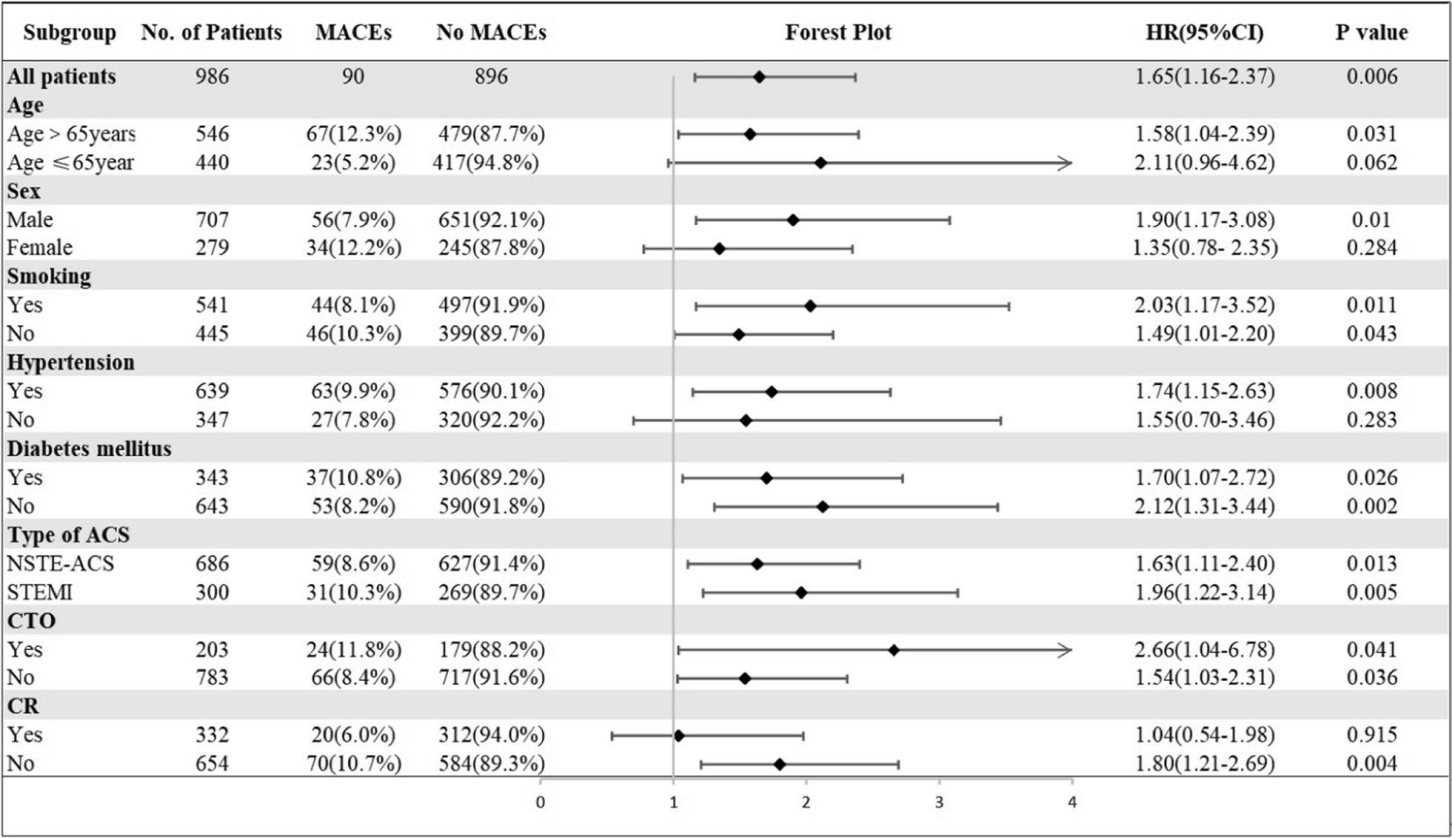
Subgroups analyses of the TyG index for MACEs. HR was evaluated by 1-point increase of the TyG index. HR, hazard ratio; TyG, triglyceride-glucose; CI, confidence interval; ACS, acute coronary syndrome; NSTE-ACS, non-ST-segment elevation acute coronary syndrome; STEMI, ST-segment elevation myocardial infarction; CR, complete revascularization, defined as residual SYNTAX score = 0

Various subgroup analyses were also performed to investigate whether the predictive value of the TyG index was similar among patients with different demographic characteristics or comorbidities (Fig. 3). We found that the TyG index was a significant predictor of MACEs regardless of current smoking, diabetes mellitus, NSTE-ACS (unstable angina + NSTEMI) or STEMI. The predictive value of the TyG index was more prominent in males, and patients with age > 65 years, hypertension, or incomplete revascularization.

### The incremental predictive value of the TyG index for MACEs

The model performance after the addition of the TyG index to the baseline GRACE score in patients with ACS undergoing PCI is presented in Tables 4 and 5. The results of the likelihood ratio tests showed that addition of the TyG index as a continuous variable significantly improved the ability of the baseline GRACE score to predict MACEs (Table 4). Addition of FBG did not improve the model fit. Comparing the baseline GRACE score alone, GRACE score with the TyG index and GRACE score with FBG, the former yielded the lowest corrected AIC compared to that with only GRACE score (Table 4). This indicate that the model with GRACE score and the TyG index is more likely to be the best fitting model compared with the other models tested.

**Table 4:** Akaike’s information criteria and likelihood ratio test to determine the best fitting model for predicting MACEs

Akaike’s information criteria					Likelihood ratio test			
Model	AICc	δAICc	AICcWt	Cum.wt	Model	χ^2^	df	P value
GRACE score	1098.73	10.35	0.01	1.00	GRACE score	Ref.	Ref.	Ref.
GRACE score + TyG index	1088.38	0.00	0.99	0.99	GRACE score + TyG index	12.37	1	<0.001
GRACE score + FBG	1100.47	12.09	0.00	1.00	GRACE score + FBG	0.27	1	0.604

MACEs indicates major adverse cardiac events, defined as a composite of all-cause death and nonfatal myocardial infarction. GRACE score, Global Registry of Acute Coronary Events score; TyG index, the triglyceride-glucose index; FBG, fasting blood glucose; AICc, corrected Akaike’s information criterion; δAICc, delta-AICc; AICcWt, AICc weights; Cum.wt, the cumulative weights of AIC

**Table 5:** Evaluation the incremental prognostic value of adding the TyG index to the GRACE score to predict clinical outcomes

	C-index (95% CI)	P value	NRI (95% CI)	P value	IDI (95% CI)	P value
*MACEs*						
GRACE score	0.735 (0.682, 0.788)	< 0.01	Ref.	Ref.	Ref.	Ref.
GRACE score + FBG	0.735 (0.681, 0.789)	< 0.01	0.068 (-0.165,0.248))	0.50	0.001 (-0.001,0.013)	0.39
GRACE score + TyG index	0.744 (0.688, 0.800)	< 0.01	0.282 (0.028, 0.426)	0.02	0.019 (0.004, 0.046)	0.01
*All-cause death*						
GRACE score	0.750 (0.690, 0.810)	< 0.01	Ref.	Ref.	Ref.	Ref.
GRACE score + FBG	0.753 (0.694, 0.812)	< 0.01	0.085 (− 0.193, 0.203)	0.41	0.002 (-0.001, 0.025)	0.39
GRACE score + TyG index	0.765 (0.704, 0.826)	< 0.01	0.308 (0.017, 0.445)	0.03	0.026 (0.004, 0.053)	<0.01
*Cardiac death*						
GRACE score	0.776 (0.708, 0.844)	<0.01	Ref.	Ref.	Ref.	Ref.
GRACE score + FBG	0.776 (0.708, 0.844)	< 0.01	0.079 (− 0.145, 0.241)	0.49	0.000 (− 0.001, 0.019)	0.52
GRACE score + TyG index	0.785 (0.714, 0.856)	< 0.01	0.336 (0.031, 0.485)	0.01	0.015 (0.003, 0.047)	<0.01
*All-cause death, MI, or unplanned revascularization*						
GRACE score	0.655 (0.609, 0.701)	< 0.01	Ref.	Ref.	Ref.	Ref.
GRACE score + FBG	0.658 (0.612, 0.704)	< 0.01	0.099 (-0.067,0.231)	0.23	0.002 (0.000, 0.009)	0.20
GRACE score + TyG index	0.673 (0.626, 0.720)	< 0.01	0.267 (0.106, 0.418)	< 0.01	0.017 (0.005, 0.035)	<0.01

MACEs indicates major adverse cardiac events, defined as a composite of all-cause death and nonfatal myocardial infarction. GRACE score, Global Registry of Acute Coronary Events score; TyG index, the triglyceride-glucose index; MI, myocardial infarction; CI, confidence interval; NRI, net reclassification improvement; IDI, integrated discrimination improvement

**Fig. 4:**
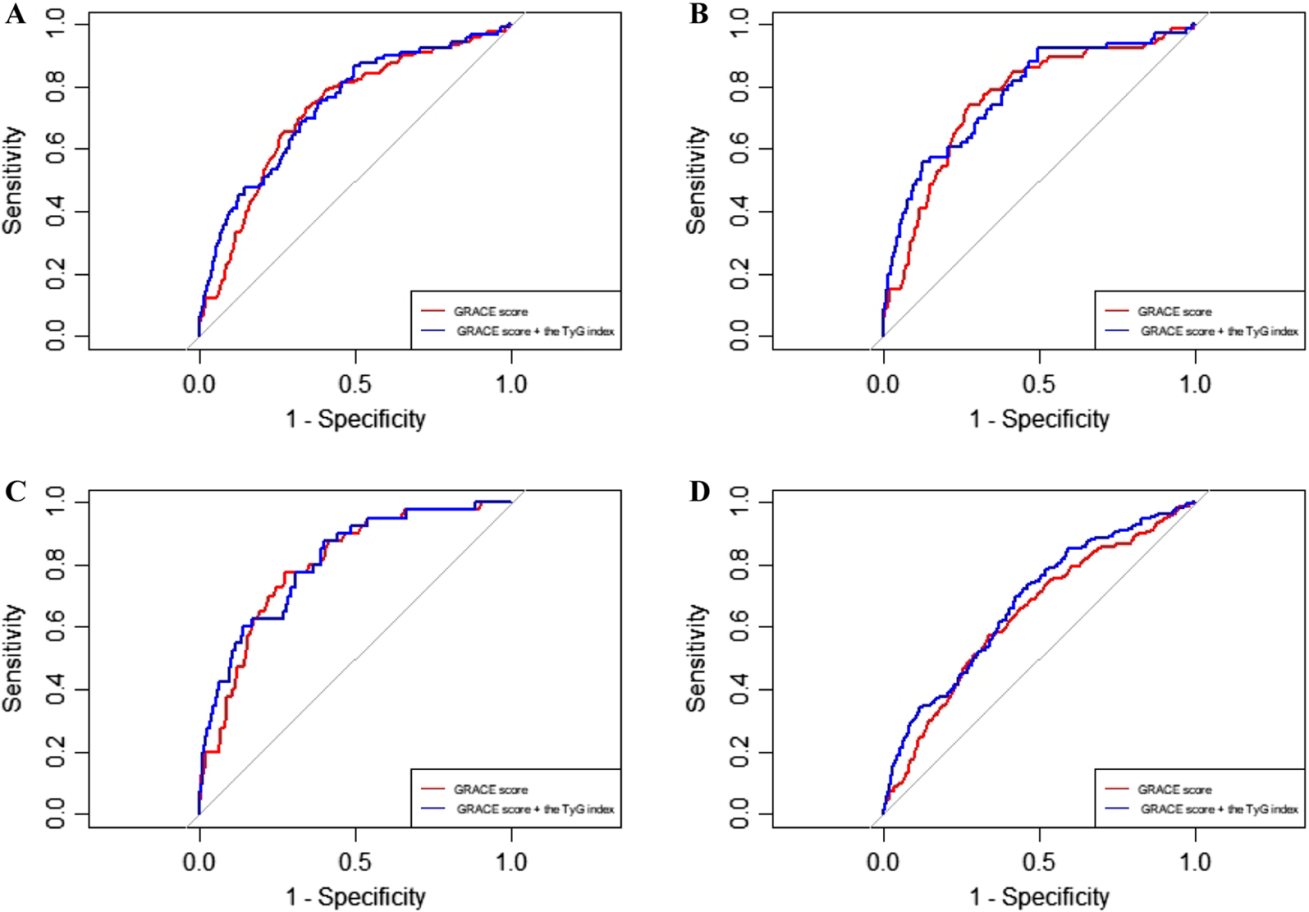
ROC curve analysis of the model performance after adding the TyG index to the baseline GRACE score. The areas under the ROC curves were used to compare the predictive ability between the baseline GRACE score and the TyG index plus the GRACE score for MACEs (**A**), all-cause death (**B**), cardiac death (**C**), and all-cause death, MI, or unplanned revascularization (**D**). ROC, receiver operating characteristic; TyG, triglyceride-glucose; MACEs, major adverse cardiac events; MI, myocardial infarction

Compared with the baseline GRACE score for predicting MACEs, the addition of the TyG index had a significant increase in the C-statistic from 0.735 (95% CI 0.682–0.788) to 0.744 (95% CI 0.6889–0.800) (P < 0.01), and significant improvement in reclassification as assessed by the NRI (0.282, 95% CI 0.028–0.426, P = 0.02) and IDI (0.019, 95% CI 0.004–0.046, P = 0.01) (Fig. 4A). Moreover, the addition of the TyG index also has an incremental predictive value for predicting other adverse cardiovascular events in terms of all-cause death (Fig. 4B), cardiac death (Fig. 4C), and all-cause death, MI or unplanned revascularization (Fig. 4D). The addition of FBG to the baseline GRACE score did not significantly improve the net reclassification and integrated discrimination in predicting events during follow-up (Table 5).

### The performance of the prediction models estimated by internal bootstrap validation

**Fig. 5:**
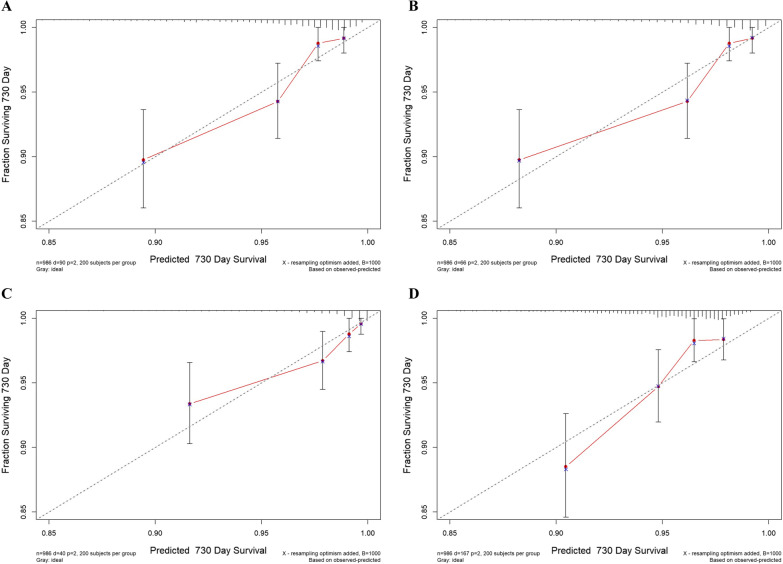
The calibration plots for adverse cardiovascular events. Calibration curves for MACEs (**A**), all-cause death (**B**), cardiac death (**C**), and all-cause death, MI or unplanned revascularization (**D**). The x-axis represents the predicted adverse cardiovascular events risk. The y-axis represents the actual adverse cardiovascular events rate. The grey line indicates a perfect prediction by an ideal model. The red solid line indicates the performance of the predicting model, of which a closer fit to the grey line suggests better prediction. MACEs, major adverse cardiac events; MI, myocardial infarction

The results remained consistent when the models were confirmed by internal bootstrap validation method (Additional file 1: Table S4). Compared with the baseline GRACE score for prediction of MACEs, the addition of the TyG index had a significant increase in the C-statistic from 0.734 (95% CI 0.683–0.787) to 0.742 (95% CI 0.688–0.796) (P < 0.01), which was significantly better than that of FBG. Trends in the same direction were observed for the prediction of other adverse cardiovascular events in terms of all-cause death, cardiac death, and all-cause death, MI or unplanned revascularization. The calibration plots for adverse cardiovascular events showed good agreement between the actual observation and the predicted possibility (Fig. 5).

## Discussion

This study shows that the TyG index, neither FBG nor TG, independently predicts prognosis after PCI in patients with ACS regardless of diabetes mellitus after adjusting for the GRACE score. The addition of the TyG index improved the ability of the GRACE score to predict long-term adverse cardiovascular events in patients with ACS undergoing PCI. Our findings support the addition of the TyG index to the model containing the GRACE score to improve the predictive value for predicting long-term adverse cardiovascular events, thus assisting clinicians in management decisions in ACS patients.

Early identification of ACS patients undergoing PCI who have a high residual risk for poor prognosis is crucial for making better management decisions to reduce future cardiovascular events. It is well acknowledged that clinical management decisions in ACS should be made on risk stratification [23]. The GRACE score has been widely accepted as a powerful predictor of adverse cardiovascular outcomes after ACS and provides validated prognostic information at different time points up to 4 years [1–3]. In line with previous research results, the GRACE score independently predicted long-term MACEs after PCI in our ACS patients, regardless of diabetes mellitus. The AUC with the GRACE score was only 0.723, which might be affected by some potential risk factors not included in the scoring model. Growing evidence demonstrates that the TyG index in the general population and in patients with coronary artery disease, regardless of diabetes mellitus is an independent prognostic factor of adverse cardiovascular outcomes [4–6, 8–13, 24]. It is worth noting that variables required for the TyG index calculation are widely available, and IR appears to be a potentially modifiable risk and therapeutic target. However, the TyG index has not been included in the GRACE scoring system in a previous study.

Several previous studies have identified some potential risk factors combined with the GRACE score, which attempted to enhance the predictive ability for adverse clinical outcomes after ACS, such as nutritional risk index [25], neutrophil count [26], B-type natriuretic peptide [27], 2-hour postload glucose [28], and hemoglobin A1c [23]. The populations enrolled in the above studies were patients with STEMI or nondiabetic patients with ACS. Whether the addition of these risk factors improves the predictive ability of GRACE score in all types of ACS regardless of diabetes mellitus was not validated. In our ACS patients, we found that both the GRACE score and TyG index were significant predictors of MACEs. Adjustment of the GRACE score by the TyG index on admission enhanced the predictive ability for MACEs after PCI in all types of ACS patients irrespective of diabetes mellitus (AUC increased from 0.723 for GRACE score alone to 0.737 for GRACE score plus the TyG index, P < 0.01). Although improving risk-prediction models containing powerful variables such as the GRACE score may be difficult, a large improvement in net reclassification was achieved (NRI, 0.282, 95% CI 0.028–0.426, P = 0.02) when adding the TyG index to the GRACE score. Our results indicate that this new risk-prediction model might be applicable to all types of ACS, which would provide a more accurate prognostic assessment in clinical practice.

Achieving complete revascularization is a desired goal of PCI in patients with coronary artery disease. Previous studies have documented that complete revascularization is more beneficial than incomplete revascularization, while residual lesions are associated with poor long-term prognosis after PCI [19, 29, 30]. However, for patients with complex coronary artery lesions, complete revascularization cannot always be achieved. In the present study, 66.3% of the patients had greater anatomic complexity and incomplete revascularization. The TyG index was an independent predictor of long-term MACEs after PCI in ACS patients with incomplete revascularization. Our findings highlight the importance of achieving a reasonable level of revascularization and providing more targeted treatment for ACS patients undergoing PCI, especially those with high residual risk for adverse clinical outcomes.

## Limitations

This study was a retrospective analysis derived from a single-center prospective observational study limited by its post hoc nature. Whether the combination of the TyG index and GRACE score improves the clinical outcomes of ACS patients undergoing PCI warrants further comprehensive investigation. The baseline level of the TyG index was derived from triglycerides and FPG on admission, which could be affected by the use of lipid-lowering and antidiabetic medications during the follow-up period. Whether the fluctuation of the TyG index impacts its predictive ability on the prognosis in patients with ACS undergoing PCI requires further investigation. Clinical trials are also needed to confirm whether improving IR can improve clinical outcomes in these patients. Finally, the results have not been externally validated. Internal validity was evaluated by the means of bootstrap with a 1000-bootstrap resampling strategy and the results demonstrated good performance in terms of discrimination and calibration for predicting risk of adverse cardiovascular events in ACS patients undergoing PCI.

## Conclusion

The TyG index was an independent prognostic predictor of long-term adverse outcomes after PCI in all types of ACS patients, irrespective of diabetes mellitus, after adjusting for the GRACE score. The TyG index improves the ability of the GRACE score to stratify risk and predict prognosis of ACS patients undergoing PCI. Whether the combination of the TyG index and GRACE score improves the prognosis of ACS patients undergoing PCI by aiding in more accurate prognostic assessment and better clinical management decisions warrants further investigation.

## Supplementary Information

**Additional file 1: **Table S1. Univariate and multivariate Cox regression analysis for predicting the primary endpoint. Table S2. The ROC curve analysis of the GRACE score, the TyG index, FBG and TG for MACEs. Table S3. The comparison of model performance. Table S4. The model performance estimated by internal bootstrap validation method.

## Data Availability

The datasets used and/or analyzed in the study are available from the corresponding author upon reasonable request.

## Abbreviations

MACEs: Major adverse cardiac events
GRACE score: The Global Registry of Acute Coronary Events score
IR: Insulin resistance
BMI: Body mass index
PCI: Percutaneous coronary intervention
COPD: Chronic obstructive pulmonary disease
AF: Atrial fibrillation
SBP: Systolic blood pressure
HR: Heart rate
BNP: Brain natriuretic peptide
FBG: Fasting blood glucos
TG: Triglyceride
TC: Total cholesterol
HDL: High density lipoprotein-C
LDL: Low density lipoprotein-C
LVEF: Left ventricular ejection fraction
ACS: Acute coronary syndrome
MI: Myocardial infarction
ACEI/ARB: Angiotensin converting enzyme inhibitor/angiotensin receptor blocker
MVD: Multivessel disease
LM: Left main disease
CTO: Chronic total occlusion
ROC: Receiver operating characteristic
TyG: Triglyceride-glucose
bSS: Baseline SYNTAX score
rSS: Residual SYNTAX score
NRI: Continuous net reclassification improvement
IDI: Integrated discrimination improvement
