# Supplementary material for: Adjustment of the GRACE score by the triglyceride glucose index improves the prediction of clinical outcomes in patients with acute coronary syndrome undergoing percutaneous coronary intervention
Source: Cardiovasc Diabetol. 2022 Aug 5;21:145. doi: 10.1186/s12933-022-01582-w (PMC9356419; doi:10.1186/s12933-022-01582-w)
Supplement: Supplementary file 1 — Additional file 1: Table S1. Univariate and multivariate Cox regression analysis for predicting the primary endpoint. Table S2. The ROC curve analysis of the GRACE score, the TyG index, FBG and TG for MACEs. Table S3. The comparison of model performance. Table S4. The model performance estimated by internal bootstrap validation method. [file 12933_2022_1582_MOESM1_ESM.docx]

**Additional materials**

**Table S1 Univariate and multivariate Cox regression analysis for predicting the primary endpoint**

| **Variables** | **Univariate analysis** | | | **Multivariate analysis** | | |
| --- | --- | --- | --- | --- | --- | --- |
|  | **HR** | **95%CI** | **P** | **HR** | **95%CI** | **P** |
| GRACE score | 1.0253 | 1.0182 to 1.0325 | <0.0001 | 1.0188 | 1.0103 to 1.0275 | <0.0001 |
| Female | 1.5832 | 1.0334 to 2.4255 | 0.0348 | 1.5257 | 0.9911 to 2.3486 | 0.0549 |
| BMI | 0.9528 | 0.8841 to 1.0268 | 0.2050 |  |  |  |
| Smoking | 0.7895 | 0.5222 to 1.1936 | 0.2623 |  |  |  |
| Previous PCI | 1.3968 | 0.7428 to 2.6268 | 0.2997 |  |  |  |
| Hypertension | 1.3202 | 0.8409 to 2.0728 | 0.2274 |  |  |  |
| Diabetes mellitus | 1.2838 | 0.8433 to 1.9544 | 0.2440 |  |  |  |
| FBG | 1.0843 | 1.0339 to 1.1372 | 0.0009 | 1.0436 | 0.9879 to 1.1024 | 0.1273 |
| TG | 1.0380 | 0.9043 to 1.1914 | 0.5961 |  |  |  |
| HDL | 0.9716 | 0.4767 to 1.9805 | 0.9368 |  |  |  |
| LDL | 0.9249 | 0.7572 to 1.1297 | 0.4441 |  |  |  |
| bSS | 1.0537 | 1.0324 to 1.0755 | <0.0001 | 1.0253 | 1.0002 to 1.0510 | 0.0479 |
| ICR | 1.9863 | 1.2077 to 3.2669 | 0.0069 | 1.2997 | 0.7589 to 2.2258 | 0.3396 |
| LVEF | 0.9448 | 0.9269 to 0.9629 | <0.0001 | 0.9763 | 0.9537 to 0.9995 | 0.0457 |
| AMI | 1.9438 | 1.2533 to 3.0148 | 0.0030 | 0.8566 | 0.5195 to 1.4123 | 0.5440 |
| β-blockers | 0.9299 | 0.5975 to 1.4473 | 0.7475 |  |  |  |
| Diuretics | 3.2982 | 2.1419 to 5.0787 | <0.0001 | 1.3626 | 0.8179 to 2.2699 | 0.2348 |
| ACEI/ARB | 1.2600 | 0.8330 to 1.9057 | 0.2737 |  |  |  |
| Insulin | 1.4863 | 0.7696 to 2.8704 | 0.2380 |  |  |  |

The primary endpoint was defined as a composite of all-cause death and nonfatal myocardial infarction. HR, hazard ratio; CI, confidence interval; GRACE score, Global Registry of Acute Coronary Events score; BMI, body mass index; FBG, fasting blood glucose; TG, triglycerides; HDL-C, high density lipoprotein; LDL-C, low density lipoprotein; ICR, incomplete revascularization; LVEF, left ventricular ejection fraction; AMI, acute myocardial infarction; bSS, baseline SYNTAX score; ACEI/ARB, angiotensin converting enzyme inhibitor/angiotensin receptor blocker.

**Table S2 The ROC curve analysis of the GRACE score, the TyG index, FBG and TG for MACEs**

| **variable** | **AUC** | **95% CI** | **specificity** | **sensitivity** | **P value** |
| --- | --- | --- | --- | --- | --- |
| TyG index | 0.607 | 0.576 - 0.638 | 62.83 | 58.89 | 0.001 |
| GRACE score | 0.723 | 0.694 - 0.750 | 65.74 | 73.33 | ＜0.001 |
| FBG | 0.586 | 0.554 - 0.617 | 57.59 | 61.11 | 0.005 |
| TG | 0.563 | 0.532 to 0.594 | 35.60 | 77.78 | 0.039 |

ROC, receiver operating characteristic; MACEs indicates major adverse cardiac events, defined as a composite of all-cause death and nonfatal myocardial infarction; GRACE score, Global Registry of Acute Coronary Events score; TyG index, the triglyceride-glucose index; FBG, fasting blood glucose; TG, triglycerides; AUC, area under the curve.

**Table S3 The comparison of model performance**

|  | **δAUC** | **95% CI** | **z statistic** | **P value** |
| --- | --- | --- | --- | --- |
| TyG index vs. FBG | 0.0211 | 0.0364 to 0.0785 | 0.718 | 0.4725 |
| TyG index vs. TG | 0.0438 | 0.00745 to 0.0802 | 2.361 | 0.0182 |

δAUC, delta-AUC, which indicates the change in area under the curve; TyG index, triglyceride-glucose index; FBG, fasting blood glucose; TG, triglycerides; CI confidence interval.

**Table S4 The model performance estimated by internal bootstrap validation method**

|  | Bias-corrected C-index (95% CI) | P value |
| --- | --- | --- |
| **MACEs** |  |  |
| GRACE score | 0.734 (0.683, 0.787) | ＜0.01 |
| GRACE score + FBG | 0.732 (0.680, 0.786) | ＜0.01 |
| GRACE score + TyG index | 0.742 (0.688, 0.796) | ＜0.01 |
| **All-cause death** |  |  |
| GRACE score | 0.750 (0.692, 0.809) | ＜0.01 |
| GRACE score + FBG | 0.749 (0.689, 0.810) | ＜0.01 |
| GRACE score + TyG index | 0.762 (0.703, 0.825) | ＜0.01 |
| **Cardiac death** |  |  |
| GRACE score | 0.775 (0.703, 0.837) | ＜0.01 |
| GRACE score + FBG | 0.771 (0.703, 0.840) | ＜0.01 |
| GRACE score + TyG index | 0.781 (0.713, 0.854) | ＜0.01 |
| **All-cause death, MI, or unplanned revascularization** |  |  |
| GRACE score | 0.654 (0.608, 0.700) | ＜0.01 |
| GRACE score + FBG | 0.656 (0.612, 0.700) | ＜0.01 |
| GRACE score + TyG index | 0.670 (0.624, 0.717) | ＜0.01 |

MACEs indicates major adverse cardiac events, defined as a composite of all-cause death and nonfatal myocardial infarction. GRACE score, Global Registry of Acute Coronary Events score; TyG index, the triglyceride-glucose index; MI, myocardial infarction; CI, confidence interval.
